# Functionalization Strategies of Non-Isocyanate Polyurethanes (NIPUs): A Systematic Review of Mechanical and Biological Advances

**DOI:** 10.3390/polym17243255

**Published:** 2025-12-06

**Authors:** Ana Velez-Pardo, Luis E. Díaz, Manuel F. Valero

**Affiliations:** 1 Master’s Program in Process Design and Management, Faculty of Engineering, University of La Sabana, Km. 7, Autopista Norte, Chía 25001, Colombia; anavepar@unisabana.edu.co; 2 Energy, Materials and Environment Group, Faculty of Engineering, Universidad de La Sabana, Chía 140013, Colombia; manuelvv@unisabana.edu.co; 3 Bioprospecting Research Group (GIBP), Faculty of Engineering, Universidad de La Sabana Campus del Puente del Común, Km. 7, Autopista Norte de Bogotá, Chía 140013, Colombia

**Keywords:** isocyanate-free polyurethanes (NIPUs), functionalization, physicomechanics properties, biocompatibility, antimicrobial activity

## Abstract

Conventional polyurethane (PU) synthesis is associated with environmental and health concerns due to the use of toxic isocyanates. In recent years, the development of non-isocyanate polyurethanes (NIPUs) has emerged as a sustainable alternative to conventional polyurethanes. However, these materials still exhibit inconsistencies in their physicomechanical and biological properties. This systematic review was conducted following the PRISMA methodology. A total of sixteen studies published between 2015 and 2025 were analyzed, focusing on functionalization techniques developed for non-isocyanate polyurethanes to evaluate their influence on physicomechanical and biological performance. The results reveal that functionalization can be achieved through the incorporation of inorganic additives, polar or ionic groups, and polymeric modifiers. Among the analyzed systems, those functionalized with azetidinium and Polyethylene glycol diacrylate (PEGDA) exhibited the most balanced performance, combining high mechanical strength, low cytotoxicity, and effective antibacterial activity. Overall, these functionalizations have demonstrated significant improvements in tensile strength, thermal stability, hydrophilicity, and antimicrobial activity, facilitating broader industrial and biomedical applications. Consequently, this review concludes that functionalization plays a pivotal role in improving the overall performance of non-isocyanate polyurethanes. It represents an effective and sustainable strategy to enhance the physicomechanical and biological behavior of these materials, supporting their development for advanced applications such as bioactive coatings, membranes, and wound dressings.

## 1. Introduction

Polyurethanes (PUs) are a class of polymers that have managed to achieve massive production, estimated at 65.5 billion dollars in 2018 and projected to exceed 105.2 billion dollars by 2025 in the global market [1]. However, this extensive PU production relies heavily on petroleum-derived feedstocks, and it is estimated that global annual petroleum consumption for the synthesis of fossil-based chemicals will reach 20% by 2050, at a time when petroleum resources are drastically depleted [2]. Therefore, the impending legislation on the use of toxic chemicals under the framework of the REACH (Regulation by the European Chemicals Agency) and EMA (European Chemicals Agency) have imposed restrictions on their use [3,4]. Polyurethanes are a family of flexible materials with both hard and soft sections that are adjustable and are mostly used in the packaging, coating, and biomedical industries due to their versatility and varied properties [4,5,6,7]. It is important to know that PUs are materials used in applications such as thermoplastics, elastomers, foams, adhesives, coatings, owing to their versatility and diverse properties [8]. Also preferred materials for wound dressings because of their excellent biocompatibility, good mechanical properties, convenient processability, and flexibility at different temperatures, including room temperature [9].

Despite their wide applicability, it is important to note that the conventional production of PUs relies on toxic isocyanates that release carcinogenic amines harmful primarily to human health, related to arthritis, chronic respiratory diseases (asthma), skin irritation (severe dermatitis), and also to the environment, because their non-biodegradability and the extensive use of petroleum-derived chemicals represent a significant environmental problem [1,5,9]. In this context, isocyanate-free polyurethanes (NIPUs) have emerged as a promising and environmentally friendly alternative, since their synthesis is carried out through a more ecological route, normally produced from bio-based resources such as amino acids, soybean oil, diglycerol, and sebacic acid, among others [5,10].

The usual way to obtain NIPUs is through the polyaddition of cyclic carbonates (CC) and the union of these with polyfunctional amines. These are simple and harmless reactions and processes [11]. Consequently, they are being rapidly researched and developed for use in thermosetting applications, such as surface coatings, elastomers, anti-corrosion materials, and biomedical applications [2]. Compared with classical PUs, NIPUs have interesting physicochemical properties such as thermal and hydrolytic properties, due to the presence of hydroxyl groups in the main chain [12]. These properties also favor polarity and stability, as well as generating greater water absorption [12]. Regardless of these inherent advantages, NIPUs still present certain limitations such as their lower molecular weight and greater dispersion that can affect their mechanical properties, this has driven the need for their functionalization [13].

However, NIPUs can present drawbacks in their mechanical properties, mainly in materials that derived from vegetables oils, due to the non-linear and asymmetric structure of the monomers [9] as the lack of specific functional activities given that some “basic” NIPUs do not inherently possess properties with biological activity, which are crucial for advanced applications [6]. To overcome these specific limitations and provide NIPUs with characteristic properties and functionalities, strategies have been developed aiming the improving biodegradability, biocompatibility, mechanical properties (tensile strength, Young’s modulus, percentage elongation, and water absorption ratio), as well as to introduce antimicrobial, anti-inflammatory, antioxidant, and thermal activities, among others [14]. These strategies involve the incorporation of different compounds or materials, such as azetidinium groups, titanium dioxide, silver nanoparticles, quaternary ammonium groups, polyethylene glycol diacrylate, graphene oxide, gelatin, chitosan, and polyhedral oligomeric silsesquioxane.

In line with this, the functionalization of NIPUs is not only proposed as a solution to overcome their mechanical and biological limitations, but also as a strategy to expand their applications in advanced areas. While there has been progress in the research and development of these materials, there are still challenges that must be addressed to improve their properties in different contexts. Accordingly, the objective of this research is to explore: How does functionalization influence the physical, mechanical, and biological properties of NIPUs, and their potential for adaptation to diverse industrial requirements? Thus, this review aims to analyze the various functionalization strategies of NIPUs and to assess their potential as high-performance materials within industries oriented toward developing more sustainable and less toxic materials for both human health and environment.

## 2. Materials and Methods

### 2.1. Search Strategy

A search of reports from 2015 to 2025 was conducted on Scopus and Web of Science databases on 11 August 2025 according to PRISMA (Preferred Reporting Items for Systematic Reviews and Meta-Analyses) guidelines. For Scopus databases the search equation used was TITLE-ABS-KEY (“non isocyanate polyurethane” OR “non-isocyanate polyurethane” OR “NIPU” OR “NIPHU” OR “polyhydroxyurethane” OR “polyhydroxyurethanes”) AND TITLE-ABS-KEY (biomaterial* OR bioactive* OR biocompatib* OR biomedical OR “medical application” OR antibacterial OR antimicrobial OR antifungal OR antioxidant OR “anti-inflammatory” OR “anti-thrombogenic”) AND NOT TITLE-ABS-KEY (foam OR adhesive OR hydrogel OR “tissue engineering”) and for Web of Science the search equation was (“NIPHU” OR “NIPU” OR (“non-isocyanate polyurethane” OR “non isocyanate polyurethane” OR “NIPU” OR “NIPHU” OR “polyhydroxyurethane” OR “polyhydroxyurethanes”) AND (biomaterial* OR bioactive* OR biocompatib* OR biomedical OR “medical application” OR antibacterial OR antimicrobial OR antifungal OR antioxidant OR anti-inflammatory OR anti-thrombogenic) NOT (“foam” OR “adhesive” OR “hydrogel” OR “tissue engineering”) “non isocyanate polyurethane” OR “non-isocyanate polyurethane”) AND (“biomaterial” OR “bioactive” OR “antibacterial” OR “antimicrobial” OR “antifungal” OR “antioxidant” OR “anti-inflammatory” OR “anti-thrombogenic” OR “medical application” OR “biomedical” OR “biocompatible” OR “biofunctional” OR “functional biomaterial”) NOT (“foam” OR “adhesive” OR “hydrogel”) The searches were restricted to original research articles.

### 2.2. Inclusion and Exclusion Criteria

The inclusion criteria were established as follows: (1) studies reporting on functionalized polyhydroxyurethanes; (2) studies evaluating biological activities, including biocompatibility, hemocompatibility, applications in tissue engineering, or assessments of physical, mechanical, and thermal properties; and (3) studies presenting results from in vitro or in vivo biological assays. The exclusion criteria were defined as follows: (1) publications not written in english; (2) brief reports lacking sufficient data; (3) review articles, systematic reviews, book chapters, or meta-analyses; (4) studies restricted to in silico approaches; and (5) studies not addressing functionalized polyhydroxyurethanes.

### 2.3. Selection and Data Collection Process

The selection of scientific information was conducted in two phases. First, three investigators independently performed a blinded screening of titles and abstracts, applying the predefined inclusion and exclusion criteria. Discrepancies among reviewers were resolved through discussion and consensus. The second phase was carried out by one researcher assessing the full text of potentially eligible articles to determine their final inclusion. For data extraction, a standardized extraction matrix was developed and reviewed by all three investigators. Data was entered and verified into the matrix by one researcher.

## 3. Results

### 3.1. Selection Process and Overview of Articles Included

**Figure 1:**
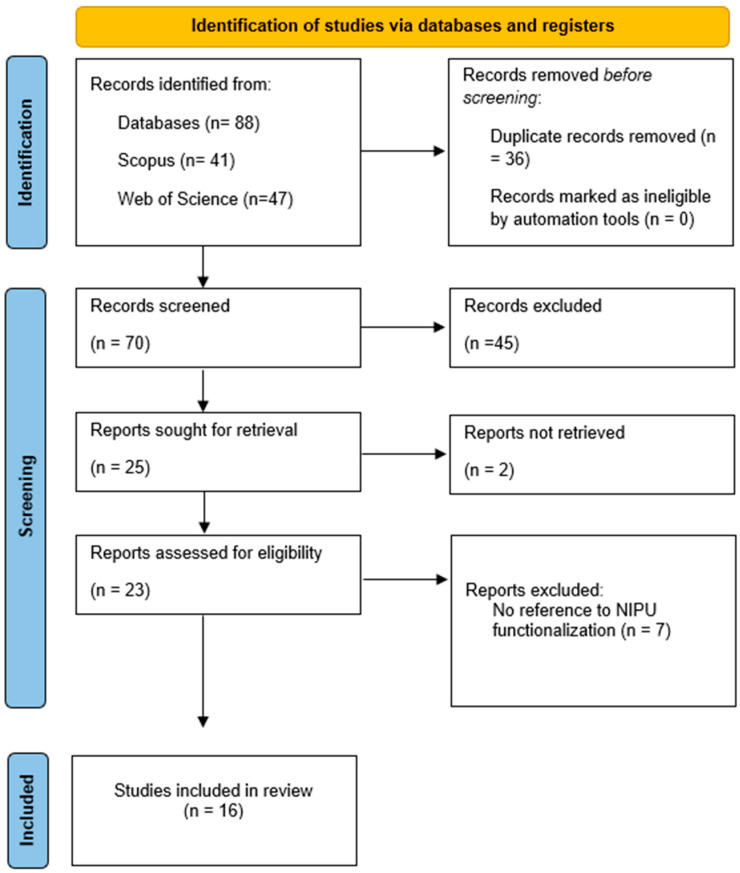
Flow diagram of the systematic literature search according to PRISMA guidelines.

After manual retrieval, the initial database search identified 88 relevant reports. Following removal of duplicates and non-English articles, 70 records were screened (Figure 1). Of these, 45 were excluded and 25 were included for full-text assessment. Finally, 16 reports met the inclusion criteria and were incorporated into the data extraction process.

**Figure 2:**
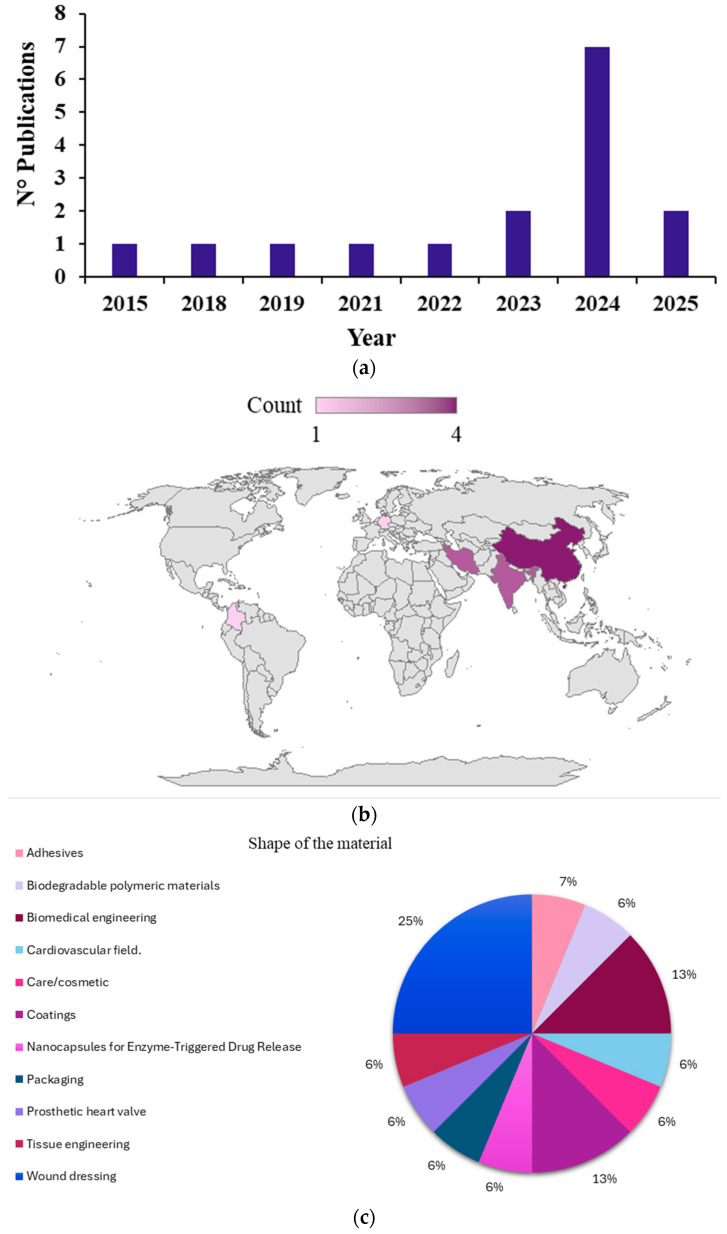
Distribution of reports on NIPUs functionalization in this systematic review: (**a**) by publication year; (**b**) by publication country; (**c**) by shape of the material.

Regarding the reports excluded after full-text examination, as shown in Figure 1, the main reason was that they addressed only non-functionalized NIPUs (*n* = 8). On the other hand, the studies included in this systematic review were published between 2015 and 2025 (Figure 2a). It is noteworthy that in 2016, 2017, and 2020 no studies were published on NIPU functionalization aimed at improving mechanical, physical, or biological properties. A steady increase in publications was observed from 2023 to 2024, reaching a peak in 2024 (*n* = 7), whereas only two studies have been published to date in 2025 (*n* = 2). Geographically, China has led the research in this field (*n* = 4), followed by Iran (*n* = 3) and India (*n* = 3) (Figure 2b). In terms of applications, wound dressings accounted for 25% (4 studies), while coatings and biomedical engineering represented 13%. Less frequently reported applications included cosmetics, packaging, the cardiovascular field, and others, each ranging between 6 and 7% (Figure 2c).

### 3.2. Functionalized NIPUs

NIPUs have been mainly obtained through the polyaddition reaction by ring-opening of cyclic carbonates with amines [15]. All the reviewed articles (*n* = 16) employed this synthesis technique, and only a few incorporated an additional step in the process; however, the underlying principle remains the same, enabling the production of high-performance, biodegradable, CO_2_-based polymeric materials with good sustainability, a low carbon footprint, and ideal biocompatibility [16]. Several carbonate sources have been explored, including bio-based carbonates such as cyclic soybean oil carbonate (CSBO) (*n* = 3) and cyclic sunflower oil carbonate (*n* = 1), as well as non-biological monomers such as propylene carbonate (*n* = 2). Understanding the synthesis process and identifying the fastest and least complex route is crucial. For instance, NIPU synthesis via transurethanization requires approximately 6–7 h to complete [17]. By contrast, the reaction of FBC monomers with diamines can be completed in just 2 h within a temperature range of 60–100 °C [16]. This method is also the simplest, as it requires neither metal nor catalysts.

The selection of amines plays a critical role in the synthesis due to their influence on the crosslinking density of NIPUs [5]. The most used amines include ethylenediamine (EDA) (*n* = 4), followed by isophorone diamine (IPDA), 1,6-hexanediamine (HDA), and 4-((4-aminocyclohexyl)methyl)cyclohexanamine (MBCHA), with two cases each. As shown in Table 1, most of the reviewed articles applied functionalization as a post-synthesis physical modification (*n* = 14) [3,4,9,11,16,17,18,19,20,21,22,23,24,25], whereas only two studies [5,8] reported chemical modification carried out during the NIPU synthesis reaction itself.

Various functionalized NIPUs have been revised for applications across different industries. Table 1 summarizes the modifications performed and the corresponding synthesis techniques employed to incorporate better mechanical, thermal, and biological properties. Moreover, both the preparation of the base material and its functionalization should be designed to remain as straightforward and optimized as possible. The minimum time required to incorporate functional properties is generally 12 h or more, as demonstrated in studies such as the integration of azetidinium groups—where the functionalized material was obtained by heating the mixture at 90 °C for 12 h [9], the biofunctionalization with rat tail collagen, in which films were incubated in the collagen solution at 37 °C for 2 h and subsequently dried at 4 °C overnight [17]. All the reviewed studies employed distinct compounds for functionalization, including titanium dioxide nanoparticles (TiO2 TNPs), azetidinium groups, polyhedral oligomeric silsesquioxane (POSS), and carboxymethyl cellulose, among others [4,5,8,9]. Notably, the most common forms of the resulting NIPU materials were films (*n* = 4) and, membranes (*n* = 3).

### 3.3. Mechanical Properties of Functionalized NIPUs

A crucial challenge in incorporating new and improved properties into NIPUs, as mentioned earlier, is achieving mechanical properties comparable to those of conventional polyurethanes (PUs) while simultaneously conferring biological functionalities. For this reason, the main physical-mechanical properties of the functionalized NIPU are established in Table 2.

It is well established that high molecular weights are required for adequate mechanical strength, as the formation of a network of secondary crosslinks increases the overall crosslinking density [22]. Accordingly, a common strategy to enhance tensile strength is to increase molecular weight; therefore, accurate characterization of this parameter is an essential step [21]. This becomes particularly relevant when NIPUs are designed for tissue engineering applications, especially when processed via electrospinning [17]. In line with this, Fan Ge [18] reported that low-molecular-weight polyglycolic acid diol (LPGD) could not be processed by electrospinning due to its low strength and insufficient molecular weight, requiring copolymerization with an NIPU to overcome these limitations.

Among the reviewed studies, the highest molecular weight (Mw) achieved was 58,600 g/mol, obtained via a transurethanization route using 1,6-HDC and PCDL500. In this case, the relatively low viscosity of PCDL500, combined with continuous stirring under reduced pressure, facilitated methanol removal and enabled the synthesis of NIPUs with the highest reported molecular weight values [17]. Conversely, the lowest Mw values (1000–5000 g/mol) were reported for phosphorylation-based functionalization. However, these results may be underestimated, since interactions between phosphonic acid polymers and the column can yield apparent molecular sizes smaller than the actual ones when compared to a neutral standard such as polyethylene oxide (PEO) [25].

The functionalization strategies for NIPUs reported in the literature and can be grouped into several categories: (i) inorganic additives (Titanium dioxide (TiO_2_) [5], POSS [8], EGO/Ag NPs [21]), (ii) ionic or polar modifications (azetidinium [9], phosphate [25]), (iii) structural reinforcements (PET mesh [3]), (iv) copolymerization with other polymers (PPG [3], PTHF [3], PEGDA [11], PCDL [17], CMC [4]), and (v) functional coatings (chitosan/alginate in LbL assembly [20], acrylic acid grafting [24], tea tree oil impregnation [20]). These strategies induce distinct effects on both physicochemical and biological properties. Based on this classification, the specific properties assessed in each study were analyzed, and the results are summarized in Table 2.

**Figure 3:**
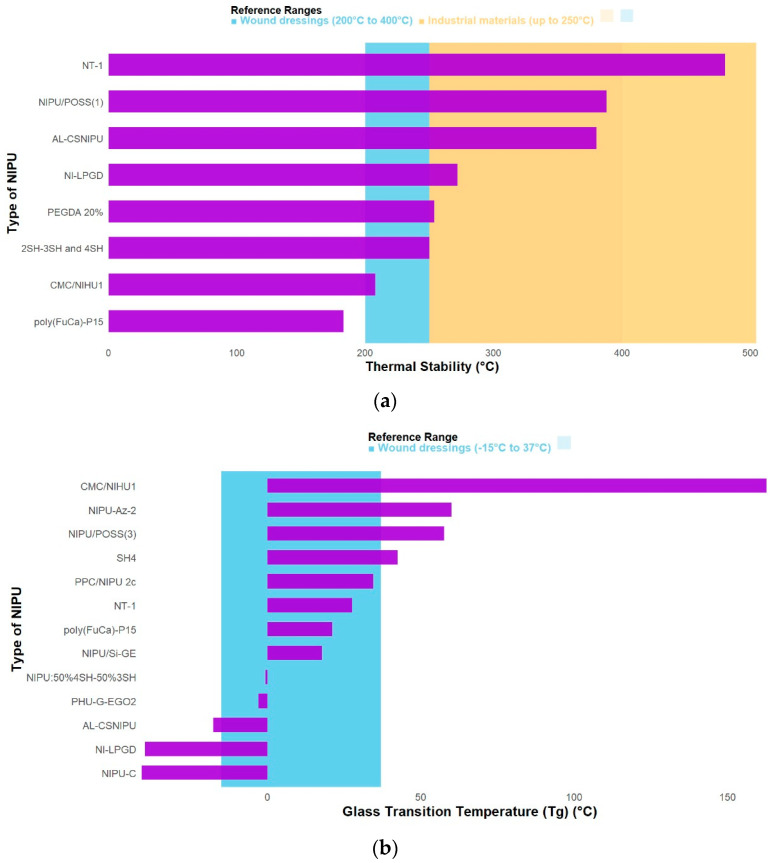
Comparison of thermal properties according to the functionalization of the NIPUs, (**a**) Thermal stability and (**b**) glass transition temperature.

Regarding thermal properties, NIPUs functionalized with POSS exhibited the greatest increase in thermal stability, reaching a decomposition temperature of 388.22 °C as can be seen in Figure 3a. This effect has been attributed to the high intrinsic stability of POSS monomers [8]. Similarly, incorporation of titanium dioxide (TiO_2_) nanoparticles (TNPs) also improved thermal stability, achieving a decomposition temperature (Td) of 480 °C (Td95%) and an initial stability of 225.87 °C (Td5%). These results were explained by the formation of tetrahedral O–Ti–O bonds within the polymer matrix through electrostatic interactions, together with hydrogen bonding between TNPs and the polymer backbone, both of which contribute to enhanced stability of the nanocomposite films [5].

In contrast, Pilar Maya [20] reported that NIPUs functionalized with tea tree oil (TTO) showed the lowest thermal stability, with an initial degradation temperature of 110.87 °C, attributed to the loss of volatile compounds from the oil incorporated on the film surface. Nevertheless, this same bioactive compound was responsible for the antibacterial activity, illustrating that certain modifications are not necessarily aimed at improving all material properties simultaneously but rather prioritize the property most relevant for the intended application (e.g., bioactivity over thermal stability in wound dressings). It is also important to note that not all studies evaluated thermal stability after functionalization [3,24]. Although the reasons for this omission were not specified, such analysis could provide valuable insights and further strengthen the research findings.

Although the thermal stability of NIPUs largely depends on the nature of the functional groups and the intermolecular interactions introduced into the material matrix [26], it is also important to analyze the behavior of the glass transition temperature (Tg), which represents the temperature at which the material transforms from a rigid glassy phase into a supercooled liquid or soft phase [27]. At this stage, it is relevant to discuss which functionalization led to a more glassy or more rubbery material, this is seen in Figure 3b. In the studies reviewed, the material classified as extremely rigid (glassy) was CMC/NIHU1, with a Tg of 162.7 °C. The study focused on whether the addition of NIPU improved the properties of CMC; in all CMC/NIHU hybrids, similar thermogram patterns were observed, indicating that the addition of NIHU to CMC did not alter its structure, and vice versa [4]. Conversely, in the opposite case, a Tg of −40 °C was reported, indicating that materials modified with LPGD exhibited good flexibility at low temperatures (below ambient conditions); however, no improvement was observed compared to the unmodified NIPU [18].

The thermal stability of a commercial wound dressing is typically within the range of 200–400 °C, involving the rupture of glycosidic bonds and the decomposition of hydroxyl groups along the polymer chain [28]. Likewise, materials used in wound dressings must remain flexible at skin temperature; thus, the Tg should be below 37 °C, generally at negative values, preferably under −15 °C [29,30]. For polyurethanes intended for industrial applications, thermal stability can reach up to 250 °C [31]. Considering these reference values, NIPUs functionalized with POSS and TiO_2_ nanoparticles exhibit thermal stability compatible with both biomedical and industrial applications, while LPGD-modified systems—with Tg values around −40 °C—show adequate flexibility for skin-contact materials.

Since crosslinking density is directly related to mechanical properties, its evaluation is essential to define an appropriate performance range. Elastic stiffness is measured through the storage modulus (E′) —that is, how difficult it is to deform the material under dynamic loads—and is also related to a polymer’s ability to store reversible energy [32]. In this context, 16% PEGDA exhibited a storage modulus of approximately 5 GPa at −75 °C. This behavior highlights the influence of temperature on stiffness, as within the range of 0–100 °C, E′ decreases sharply due to the transition from the rigid to the rubbery state. This material also presented the highest crosslinking density, confirming the critical role of crosslinking degree in determining storage [11].

**Figure 4:**
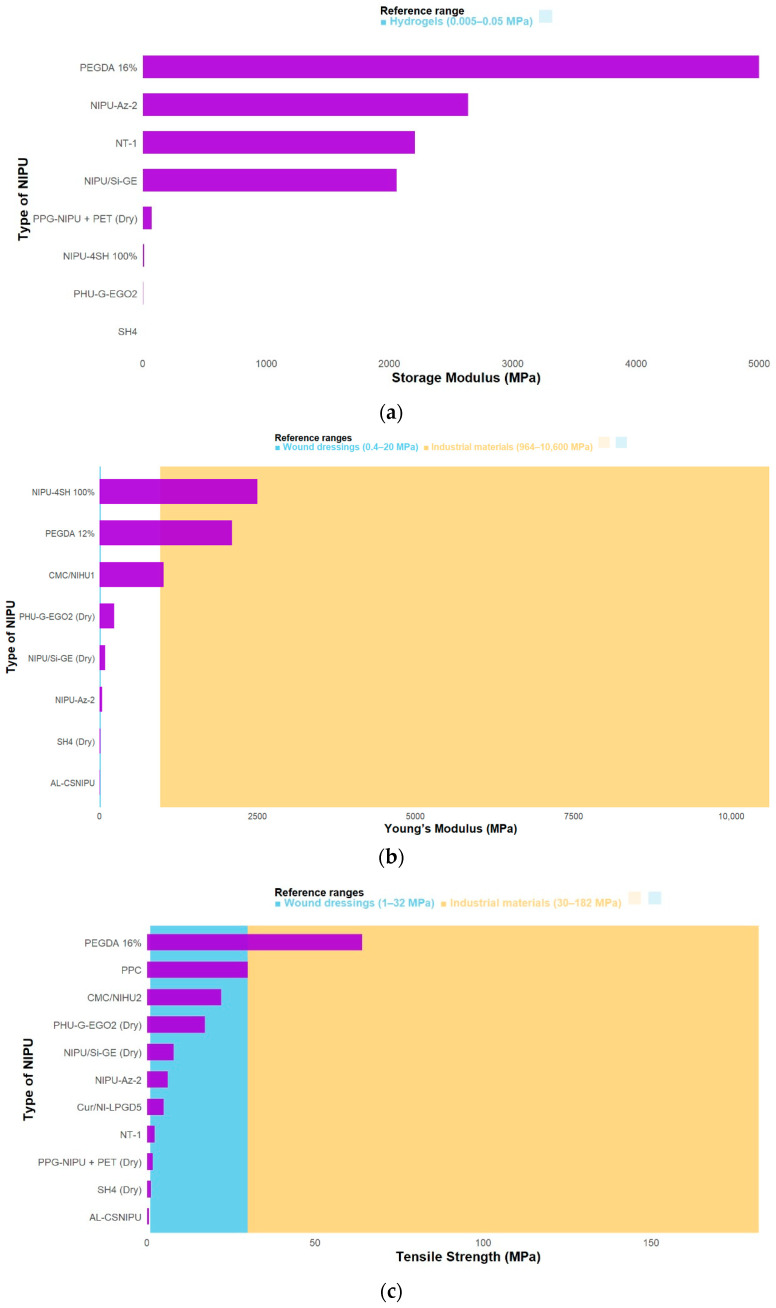

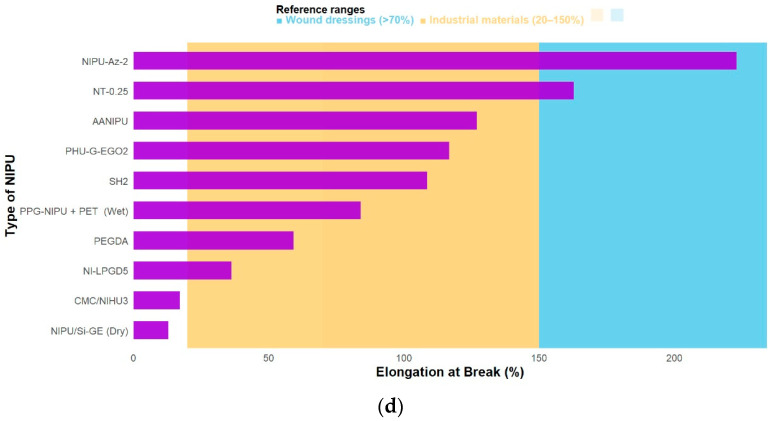
Comparison of mechanical properties according to the functionalization of the NIPUs, (**a**) storage modulus, (**b**) Young’s modulus, (**c**) tensile strength, (**d**) elongation at break.

Similarly, modifications with azetidinium groups demonstrated excellent performance, as can be seen in Figure 4a. These groups are obtained through the reaction of a secondary amine with epoxide, producing the corresponding azetidinium compound, typically with high yield and purity [33]. In this case, an E′ modulus of 2638.6 MPa was achieved in the glassy state, indicating enhanced stiffness at low temperatures, attributed to increased physical crosslinking. This effect arises from the ionic interaction of azetidinium groups formed in the polymer chain through the partial reaction of secondary amines with epichlorohydrin (ECH). Moreover, this reaction generates additional C–N crosslinks, further enhancing the mechanical strength of the resulting NIPUs [9]. On the other hand, some functionalization strategies reduce rigidity. A representative example is the thiol-ene polymerization of alkene groups. The NIPU-4SH 100% sample, with a value of 1.2E Pa, exhibits the lowest rigidity of all the articles analyzed (with the highest value among all thiol group ratios being 50% 4SH–50% 2SH at 2.0E Pa). This occurs because a quasi-crystalline structure, or more precisely, more ordered packing, is formed, which results in a relaxation of the network, thus reducing rigidity [23].

In line with what has been discussed regarding a material’s ability to store elastic energy under load [34], it is also necessary to consider the relationship between tensile stress and tensile strain in the elastic regime, which is evaluated through Young’s modulus (Figure 4b) [35]. The modification through thiol-ene polymerization of alkene groups carried out by Warner resulted in a modulus (E) of 2500 MPa. This occurs because, as the concentration of the crosslinking agent increases, the force required to elongate the polymer film decreases [19]. Interestingly, the next material with an optimal modulus (E) was the NIPU containing 12% PEGDA. The presence of long chains contributes to a more robust network structure and superior mechanical properties; however, when the PEGDA content exceeds 12 wt%, these properties are adversely affected by [11].

The use of polysaccharides such as alginate and chitosan (AL–CS) leads to a decrease in modulus due to the presence of a thin, fragile, and heterogeneous AL–CS coating network. In this case, a modulus (E) of 7.4 MPa was obtained, even lower than that of the NIPU synthesized sunflower oil [20]. This highlights the continued effort to identify compounds that can enhance desired properties without compromising the intrinsic characteristics of NIPU films.

Among the most relevant parameters is the tensile strength (Figure 4c), defined as the maximum stress a material can withstand before failure [36]. In fact, it is one of the key analyses used to determine whether a material is mechanically resistant [36]. This property often follows a similar trend to Young’s modulus, since both depend on the material’s crosslinking density. Accordingly, the PEGDA-functionalized NIPU exhibited the highest tensile strength, whereas the AL–CS-functionalized material showed the lowest [37]. Incorporating 16% PEGDA into the NIPU resulted in a tensile strength of 63.93 MPa, since longer chains reinforce the polymer structure [11]. The lowest value was observed for the NIPU functionalized with alginate and chitosan (AL–CS/NIPU), with a tensile strength of 591.99 ± 130.3 kPa. The stiffness of this material is influenced by the highly rigid chitosan chains compared with the more flexible NIPU or alginate chains [20].

Lastly, among the mechanical properties, elongation at break is one of the most relevant parameters. Also known as fracture strain, it represents the ratio between the modified length and the initial length after the specimen fails. Like other mechanical parameters, it reflects whether the material behaves in a more rigid or elastic manner [38]. In Figure 4d it can be observed that the modification that resulted in the highest elongation at break was the incorporation of azetidinium groups, namely in NIPU-Az-2, which exhibited an elongation of 223 ± 7%. Despite being the highest value among all the studies reviewed, this functionalization slightly decreased the elongation compared to the unmodified NIPU. This reduction is attributed to the partial consumption of the primary amine groups in TEPA and the consequent decrease in the chemically crosslinked density [9].

Conversely, the stiffest material was the one modified with CPTMS, GPTMS, TEOS, and gelatin, which achieved an elongation of only 12.90%, making it the material with the lowest ductility. This decrease can be attributed to the formation of a secondary crosslinked network; in addition, the presence of gelatin introduces additional hydrogen bonds and van der Waals interactions, both of which contribute to the increased stiffness, as previously discussed [22].

An ideal wound dressing requires a combination of high elongation at break, high tensile strength, and a low Young’s modulus to ensure durability and resistance to stress during application and handling. For these materials, tensile strength typically ranges from 1 to 32 MPa, elongation at break should exceed 70%, and the Young’s modulus is expected to fall between 0.4 and 20 MPa, values that correspond to the mechanical behavior of native human skin [39]. Although storage modulus values are more frequently reported for hydrogels than for polymeric films, standard ranges for these systems lie between 5000 and 50,000 Pa [40].

In contrast, for industrial applications, polyurethanes exhibit considerably higher performance requirements, with tensile strength values ranging from 30 to 182 MPa, elongation between 20% and 150%, and Young’s modulus values spanning from 964 to 10,600 MPa, depending on the specific end use, including automotive components, electronic devices, and household equipment [41]. None of the functionalized materials analyzed fully meet the ideal ranges for commercial wound dressings, although some (such as NIPU-Az-2 and PHU-G-EGO2) partially approach these parameters. In contrast, those exhibiting higher rigidity (PEGDA and thiol-ene systems) would be more suitable for industrial applications that require high mechanical strength.

Based on the above and on the observations in Table 1 and Table 2, it can be interpreted that the thermal and mechanical variations originate from the different functionalization strategies, as these modify the segmental mobility and the crosslinking density of the matrix [5,22,42]. This directly affects not only Young’s modulus, tensile strength, and elongation at break—as evidenced in the reviewed articles [3,4,5,6,9,19,20,21,22,23]—but also, by promoting crosslinking reactions and the formation of additional covalent bonds [43].

Depending on the intended application, certain material properties are prioritized over others [44]. For instance, systems modified with inorganic compounds such as TiO_2_ reinforce the structure by forming rigid domains through O-Ti-O bonds, while POSS (Polyhedral Oligomeric Silsesquioxane)—owing to its high intrinsic stability [8]—prevents nanoparticle regrouping, thereby enhancing thermal performance, particularly the glass transition [11,45]. In both cases, restricted mobility is the common mechanism, as the hydroxyl groups present form hydrogen bonds with the NH groups of the polymer backbone; these interactions restrict chain mobility [5], suppress chain transfer reactions during thermal degradation, and increase decomposition temperatures [5,46].

Following this premise, when the objective shifts toward materials with greater strength and stiffness, mechanical properties must take precedence over other factors. The observed patterns confirm that crosslinking density is the primary factor explaining variations in mechanical performance [47]. Highly crosslinked systems—such as the one formulated with 12 wt.% PEGDA [11]—exhibit high modulus and strength values due to the presence of long chains capable of generating robust networks; however, crosslinking must also be sufficiently homogeneous to allow an adequate number of effective chains and stable support under tension, thereby reducing the propagation of stress between fragments [11,48].

Similarly, ionic groups such as azetidinium introduce reinforcement through physical crosslinking, derived from ionic interactions between groups formed in the polymer backbone because of the partial reaction between secondary amines and ECH, contributing additional strength along with the formation of secondary C-N bonds [9]. In direct contrast, formulations based on thiol-ene reactions can drastically decrease stiffness when the network acquires a more crystalline and ordered structure, facilitating packing relaxation and reducing the modulus to the lowest values within the analyzed systems [19]. These findings demonstrate that not all crosslinking uniformly improves mechanical properties: when crosslinking density is prioritized, maximum stresses and rupture percentages increase under tension and shear, while maximum strains decrease [47].

Reinforcements based on biopolymeric compounds such as alginate (AL), chitosan (CS), and carboxymethylcellulose (CMC) exhibit differentiated behaviors influenced by the chemical and physical microstructure of each polymer [49]. AL-CS systems produce brittle films with low modulus and strength due to the intrinsic stiffness of chitosan and the formation of a brittle and heterogeneous surface network, which results not only in poor tensile strength but also in a drastic reduction in flexibility [20]. Conversely, CMC/NIHU hybrids display the opposite behavior; CMC shows good interfacial compatibility and homogeneous integration into the network, generating multiple interaction points through hydrogen bonds that reinforce internal cohesion and lead to significantly higher Young’s modulus and tensile strength values than in the previous case [4], placing them among the systems with the best reported mechanical performance.

This evidence allows us to affirm that a highly crosslinked surface network reinforced through various inter and intramolecular interactions such as ionic bonds and hydrogen bonds can lead to extreme rigidity [20], while also affecting other functional properties of material. Therefore, this type of reinforcement must be selected precisely according to the intended application [44]. Taken together, these results confirm that the final mechanical performance does not depend solely on the additive itself, but rather on how it reorganizes the NIPU microarchitecture, modifying the crosslinking density, packing, and chain mobility, which in turn defines differentiated routes toward biomedical applications or toward rigid materials for industrial use [5,9,19,20,41].

### 3.4. Physical Properties of Functionalized NIPUs

While it is true that NIPUs must exhibit adequate physical and mechanical properties for every intended application, wound dressings require physical characteristics comparable to those of human skin, which makes the development of a fully suitable material more challenging [18]. Therefore, it is essential to study and evaluate physical parameters such as water absorption, contact angle, and water vapor transmission rate (WVTR). Even when the final application is not biomedical, the values of these specific properties are presented in Table 2 (Improved physical and mechanical properties of functionalized NIPUs).

**Figure 5:**
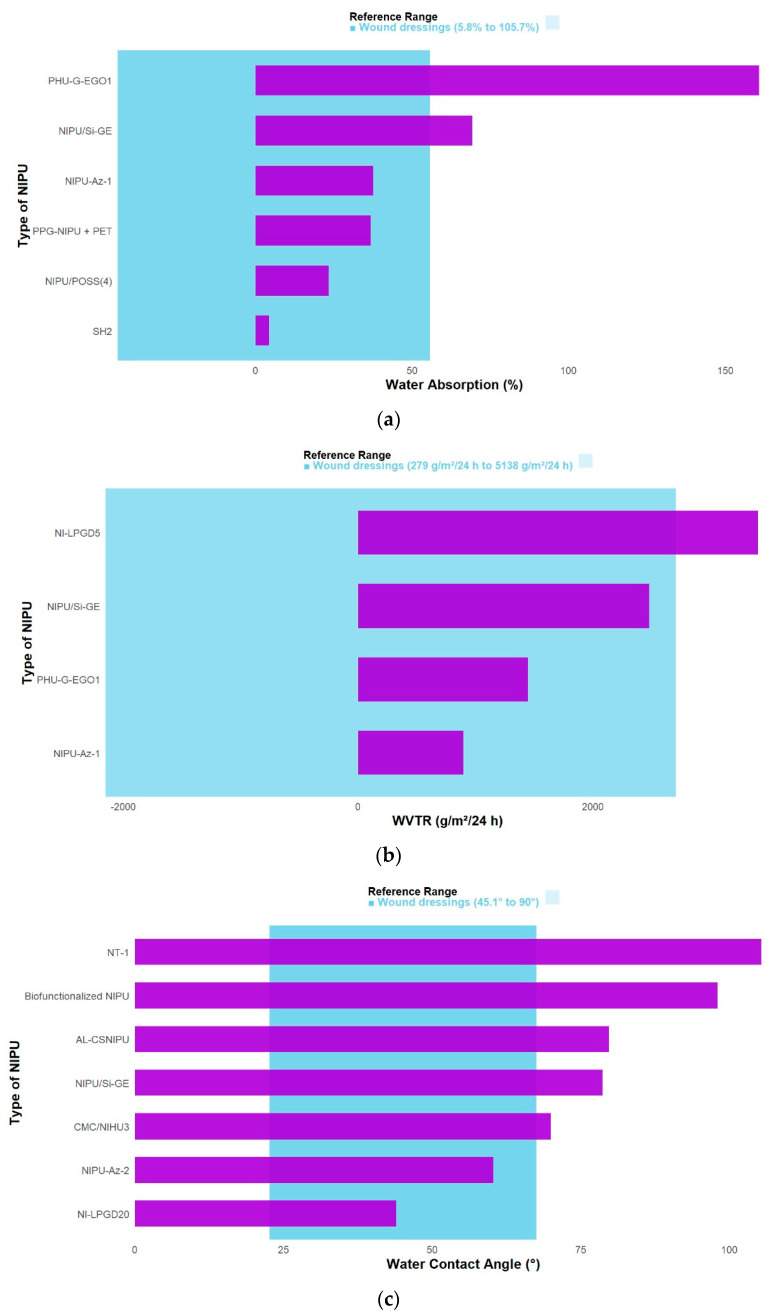
Comparison of physical properties according to the functionalization of the NIPUs, (**a**) absorption of water, (**b**) water vapor transmission rate, (**c**) water contact angle.

Water absorption is defined as the amount of water absorbed by a sample under specific conditions, typically upon immersion in water [50]. The functionalization showing the highest EWA% (equilibrium water absorption) corresponds to NIPU PHU-G-EGO1 (gelatin–epoxidized graphene oxide), with a value of 160.87%. This behavior is attributed to the presence of Poly (ethylene glycol) (PEG) and gelatin (GE) in the composition, which promote hydrogen bonding between the polar groups available in the dressing matrix—such as hydroxyl, carboxyl, amine, ether, and urethane—and water molecules [21]. Conversely, the lowest water absorption capacity was observed for the NIPU designated as SH2, which was modified with α-alkylidene cyclic carbonate (αCC) and crosslinked via the thiol-ene reaction. The water uptake in this case was only 4.2%, not only due to the hydrophobic nature of the PPG segments but also because water absorption decreases with increasing crosslinking density, which is consistent with this result [23]. Therefore, the evaluation of the amount of water that passes through a barrier per unit area and unit time—typically measured under controlled temperature and humidity conditions—is expressed as the water vapor transmission rate (WVTR), shown in Figure 5b. WVTR is the most used metric to represent the effectiveness of a moisture barrier [51]. Interestingly, many studies did not assess the WVTR; thus, the discussion and comparison in this section are based on only four investigations, as summarized in Table 2.

A WVTR value of 3405 g/m^2^·day was achieved, which was directly related to the crosslinking density of the samples. The WVTR increased as the crosslinking density decreased. This behavior suggests that the larger internal volume of the sample with the lowest crosslinking density (PHU-G-EGO2) enhanced water vapor transmission through the membrane, despite its lower capacity to absorb water molecules [21]. In contrast, functionalization with azetidinium groups yielded the lowest WVTR, with a value of 896 g/m^2^·day [9]. Although this is the lowest among the reported values, it is not considered unfavorable, as commercial synthetic wound dressings typically exhibit WVTRs ranging from 34 to 11,000 g/m^2^·day [52]. This result is explained by the ability of the network’s structural units to expand or contract in response to ambient humidity, allowing the prepared membranes to self-regulate water vapor transport according to the level of wound to exudate. Combined with their ability to retain water molecules (swelling) within their volume, these membranes help maintain an optimal moist environment [9].

In recent years, contact angle measurement has been recognized as one of the most reliable methods to evaluate the hydrophilic or hydrophobic nature of surfaces [53,54]. Films are classified as hydrophilic when the contact angle is <90°, and hydrophobic when it is >90° [46]. Based on this, in Figure 5c, the most hydrophilic material evaluated was NI-LPGD20, with a contact angle of 44°, attributed to residual fluorine present in NI-LPGD. This fluorine likely results from the secondary reaction between the solvent (HFIP) and sebacyl chloride during NILPGD synthesis, suggesting that the introduction of LPGD can enhance the hydrophilicity of the material [18]. Conversely, the material functionalized with TiO_2_ nanoparticles (TNPs) was the most hydrophobic, with a contact angle of 105.4°. This behavior is attributed not only to the long alkyl chains of soybean oil-derived triglycerides, which naturally repel water, but also to the increased nanometric surface roughness induced by the presence of TNPs [5].

In summary, the functionalized NIPUs analyzed exhibit physical properties that approximate those of commercial wound dressings. The reported WVTR values (896–3405 g/m^2^·day) fall within the optimal range for wound environments (279–5138 g/m^2^·day). Likewise, water absorption values between 4.2% and 160.87% align with the general range of 5.8–105.7%, while contact angles (44–105.4°) correspond to those of commercial dressings, typically above 45.1° but below 90° [55]. These findings confirm that appropriate functionalization enhances the hydrophilic balance and permeability of NIPUs, making them suitable for biomedical use, whereas in industrial applications, the adjustment of polyurethane feedstocks can yield materials tailored for diverse functionalities [56].

Building upon these observations, a deeper examination of the functionalization methods reveals that the physical behavior of NIPUs—particularly water absorption, vapor permeability, and contact angle—depend primarily on the crosslinking density, the degree of polarity introduced into the matrix, and the intermolecular forces between the polymer chains [26,57].

Consequently, for the design of materials requiring high hydrophilicity, high water absorption, and good vapor permeability (like PHU-G-EGO1), it is essential to prioritize the simultaneous incorporation of hydrophilic groups such as PEG and GE, whose high polarity promotes interaction with water molecules through multiple hydrogen bonds. This effect is further enhanced when the matrix contains other polar functional groups—such as hydroxyl, carboxyl, amine, ether, and urethane—which increase water-absorption capacity and explain the maximum EWA% (equilibrium water absorption) values observed [21,22]. In parallel, a lower crosslinking density increases WVTR by leaving a larger fraction of internal volume available for the formation of diffusion channels that facilitate vapor transport through the material [21].

In contrast, if the objective is to obtain materials with low water absorption and markedly hydrophobic behavior, as in the SH2 strategy based on α-CC and PPG, it is necessary to select compounds whose chains exhibit a pronounced nonpolar character in order to reduce the material’s surface energy [23]. In this context, PPG contains an additional methyl group per monomer in its polymer chain, making it more hydrophobic than other polyols and causing a rapid decrease in water solubility [58]. Furthermore, the conversion of hydrophilic hydroxyl groups into hydrophobic carbonate fractions—followed by additional chain crosslinking—further reduces the system’s hydrophilicity and significantly modifies its physical properties [23].

Taken together, these differences demonstrate that the dominant factor governing physical properties is not the mere presence of a functional compound, but its ability to alter the balance between polarity and crosslinking density [21,23,58]. The contact angle directly determines the hydrophilic or hydrophobic character of the material. This relationship is illustrated in the TiO_2_-NIPU system, which exhibits a characteristically hydrophobic contact angle of 105.4° [5]. This hydrophobicity is attributed both to the long alkyl chains of the soybean-oil derivative—naturally hydrophobic—and to the increased nanometric roughness generated by the dispersion of inorganic nanoparticles, which reduces the material’s wettability [5]. This duality shows that chemically distinct modifications can produce similar or opposite effects on the final properties depending on how they alter the structure [59].

### 3.5. Biologicals Properties of Functionalized NIPUs

If a material is intended for direct contact with blood, several key endpoints must be evaluated for cell–material interactions, including cell viability, adhesion, and spreading [60]. Therefore, NIPUs must be capable of interacting with host cells without inducing systemic or local cytotoxicity, mutagenesis, carcinogenesis, allergic responses, irritation, or inflammation [61]. According to ISO 10993-5 [62], cell viability values above 80% are classified as non-cytotoxic; between 80% and 60%, as weakly cytotoxic; between 60% and 40%, as moderately cytotoxic; and below 40%, as strongly cytotoxic [63].

**Figure 6:**
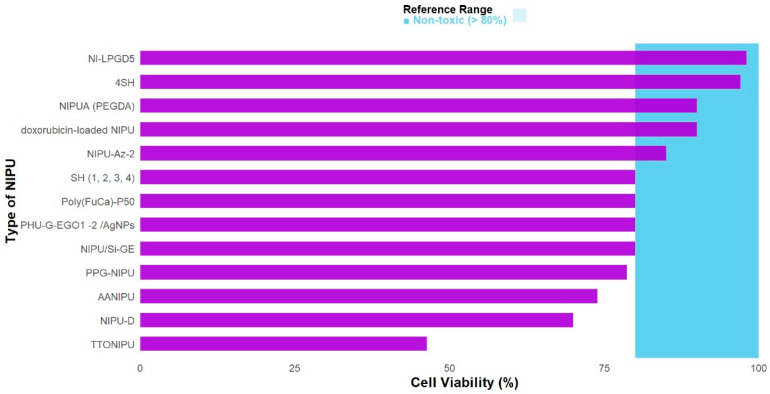
Cell viability of functionalization of NIPUs.

In this review, most of the modified NIPUs exhibited cell viability above 80%, indicating non-cytotoxic behavior, as shown in Figure 6. This is primarily attributed to the removal of toxic contaminants [19] and the adequate elimination of residual solvents, for instance through vacuum drying [17]. It also encompasses the selection of safe and biocompatible raw materials, such as gelatin—a biopolymer with a high biocompatibility index, low cytotoxicity, and the capacity to support cell adhesion and proliferation [21]—as well as hydrophilic substrates (GE, CMC, LPGD) which have been shown to enhance cell adhesion, reduce rejection, and promote direct interaction with the wound site [18].

Only one of the evaluated materials showed a viability of 46.32%, rendering it unsuitable for cell contact [TTO]. These results highlight an important contradiction; oils that are beneficial on their own can produce opposing effects when immobilized within a polymeric matrix [64,65]. This occurs because their incorporation influences not only protein adsorption but also induces surface energy changes that hinder cell interaction and the subsequent cellular response, ultimately making them cytotoxic to different cell lines [20]. Various cell types were used for viability assays, including L929 mouse fibroblasts, human primary fibroblasts, murine fibroblasts, and human keratinocyte HaCaT cells, among others. Detailed data for this biological property are presented in Table 3.

Analysis of the observed behavior among functionalized systems suggests that the biological responses of NIPUs—particularly cytocompatibility and cell adhesion—are strongly correlated with the surface properties of the biomaterials. These properties are influenced by the type of functional groups, surface charge, topography, and wettability [66]. Therefore, regardless of their origin, if the functional groups can modify these parameters, key phenomena such as cell adhesion, protein stability, and activation of initial proliferation pathways are enhanced [67]. As with other properties, biocompatibility does not arise from the functionalization strategy itself, but from how its chemistry reorganizes the surface and modulates the pathways involved in cell proliferation and adhesion [68,69].

Beyond cytocompatibility, antimicrobial activity is also a crucial factor, not only for materials intended for biomedical applications but also for coatings, films, and tissue engineering scaffolds. Antimicrobial compounds inhibit the growth of bacteria, fungi, viruses, and protozoa through specific mechanisms [42]. The relevance of these mechanisms becomes particularly clear when considering the current global challenges associated with microbial resistance. According to the World Health Organization, antibiotic resistance has become a critical global health concern, underscoring the importance of developing materials with intrinsic antimicrobial properties that do not promote long-term bacterial resistance [43]. Several of the functionalization strategies described for NIPUs imparted antimicrobial activity to the materials. However, direct comparison among studies is challenging, as the results are reported in non-standardized units, such as inhibition zone diameter (mm) or inhibition percentage (%). Despite this limitation, two functionalizations stood out for their strong antibacterial potential in vitro.

The first and most promising was functionalization with azetidinium groups, specifically NIPU-Az-2. In this study, *E. coli* ATCC 25922 and *S. aureus* ATCC 6538 were used, yielding inhibition percentages of 100% and 98.27%, respectively. This effect was attributed to the presence of quaternary azetidinium moieties generated in the polymer backbone through reaction with ECH. These moieties impart a permanent positive charge to the polymer matrix, enabling electrostatic interaction with negatively charged bacterial membranes, ultimately leading to bacterial cell death [9]. As shown in Table 3, another functionalization with high antibacterial performance was the PHU-G-EGO_2_/AgNPs system, incorporating epoxidized graphene oxide and silver nanoparticles. Against *E. coli* (strain not specified) and *S. aureus* ATCC 6538, inhibition percentages of 89.3% and 92.8% were achieved, respectively. These nanoparticles can continuously release silver ions that adhere to the cell wall and cytoplasmic membrane, increasing cytoplasmic membrane permeability and ultimately disrupting the bacterial envelope. They also inhibit protein synthesis by denaturing ribosomes in the cytoplasm and disrupting signal transduction, leading to cell apoptosis. Furthermore, the increased activity in this case is attributed to the mutual presence of GO and AgNPs, where the GO nanoplates act as “cutters” that damage and rupture bacterial membranes [22].

Although both functionalization strategies achieved outstanding antibacterial activity, one outperformed the other. This difference arises because, although AgNPs release antimicrobial ions, their performance is hindered by inherent limitations such as oxidation and aggregation, which reduce their active surface area and consequently diminish their antimicrobial efficiency [70]. In contrast, azetidinium groups act on any bacteria that comes into contact with the surface [9].

Furthermore, when these systems were evaluated against non-functionalized NIPUs, it was consistently observed that materials either lacked antimicrobial activity or exhibited only negligible effects [5,18,23,25]. This finding reinforces that the antimicrobial performance of NIPUs relies on the incorporation of specific functional groups capable, as of interacting with, disrupting, or compromising microbial structures, as the native backbone of non-functionalized NIPUs is generally biologically inactive.

### 3.6. Main Applications and Results of Functionalized NIPUs

The most widely explored application of functionalized NIPUs is wound dressings. A total of four studies classified them as suitable materials for this purpose. The main results of each of the studies and limitations can be found in Table 3.

In line with the above, the results clearly demonstrate that the overall performance of NIPUs is determined by the specific nature of the modifications introduced into their structure [20,25]. The development of multifunctional systems capable of achieving a suitable balance among biocompatibility, mechanical performance, processability, and flexibility at room temperature remains essential to achieve competitive performance compared to conventional polyurethanes [9]. This balance can be attained through the integration of a densely cross-linked network containing polar or ionic functional groups. Such modifications should promote ionic and hydrogen-bonding interactions that reinforce the polymer matrix, thereby enhancing mechanical strength without compromising flexibility or cellular compatibility [4,8,9,22].

As observed, reinforcements with polysaccharides such as alginate and chitosan generally reduce mechanical strength, preventing the attainment of the desired equilibrium in these systems [20]. Based on the evaluated properties, NIPUs functionalized with azetidinium groups and those cross-linked with PEGDA emerged as the most promising candidates, exhibiting not only excellent physicomechanical performance but also low cytotoxicity and strong antimicrobial activity—features particularly advantageous for biomedical uses such as antibacterial coatings or wound dressings [9,11]. The incorporation of bio-derived monomers, green crosslinkers, and advanced functional agents represents a promising pathway toward the next generation of NIPUs with tailored properties for biomedical and industrial applications [6,17,25].

Considering the scenario described, a critical reflection on the methodological limitations identified throughout the reviewed articles is necessary. Barriers to validating the real-world applicability of functionalized NIPUs are evident, as summarized in Table 4. Most studies rely exclusively on in vitro assays without incorporating in vivo evaluations to corroborate their findings, even when the proposed application clearly requires them, as in the case of wound dressings [71]. Furthermore, inconsistencies exist in the breadth of the evaluations, as several studies omit key properties such as Young’s modulus, tensile strength, chemical resistance, enzymatic degradation, or long-term stability—particularly in works centered on antimicrobial or coatings applications [72]. Likewise, many formulations explicitly intended for biomedical uses do not include hemocompatibility tests, advanced cytotoxicity assays, or in vivo studies, preventing the determination of their true behavior under physiological conditions and limiting the extrapolation of results to clinical scenarios [73,74].

Another recurring limitation in the reviewed literature is the absence of appropriate experimental controls—for example, comparing hybrids only against CMC rather than against pure NIPU [4]—or working with a small number of replicates, which restricts the statistical robustness of the findings [75]. Therefore, it is concluded that compliance with essential specifications, such as non-toxicity, controlled degradability, and adequate mechanical strength aligned with the characteristics of the target tissue, remains an area requiring greater attention [9].

This methodological fragmentation generates apparent contradictions between studies, as materials labeled as “promising” frequently present experimental gaps that prevent them from genuinely qualifying as fully functional candidates [76]. For this reason, it is recommended that future work incorporate comprehensive characterization aligned with current international protocols and standards (such as ISO or ASTM).

Furthermore, the studies reviewed present barriers to the reproducibility and scalability of functionalized NIPUs. All formulations have been synthesized exclusively at the laboratory scale [3,4,5,8,9,11,16,17,18,19,20,21,22,23,24,25]. Many of them require prolonged reaction or post-functionalization times (>6–12 h) [5,9,11], which hinders their scalability to continuous industrial manufacturing processes [77]. Likewise, several studies rely on fluorinated solvents (HFIP) or solvents of toxicological concern such as NMP (N-Methyl-2-pyrrolidone) or DMF (N,N-dimethylformamide) [9,17,22,23]—classified by the FDA as incompatible with safety, and sustainability requirements for industrial environments [78]. These aspects indicate that the functionalized NIPUs analyzed in this review should still be considered as materials in an exploratory phase. It is therefore suggested that future studies incorporate validation under industrial conditions to bridge the gap between their performance at the laboratory scale and their feasibility in commercial applications.

## 4. Conclusions

As highlighted in this review, although the synthesis of isocyanate-free polyure-thanes was first reported in 1957, the functionalization of NIPUs to enhance their physical, mechanical, and biological performance has only recently gained research momentum, with significant progress achieved since 2015. Nevertheless, the number of published studies remains limited compared to conventional polyurethanes, revealing a technological and scientific gap that still needs to be addressed.

The compiled evidence confirms that the incorporation of specific functional groups can overcome several intrinsic limitations of NIPUs, such as low mechanical strength, restricted structural flexibility, and limited bioactivity. Among the strategies analyzed, the introduction of azetidinium groups stands out for providing the most favorable balance between mechanical robustness, cytocompatibility, and antimicrobial activity, positioning this approach as a promising route for the design of wound dressings and bioactive coatings. However, none of the systems reviewed simultaneously fulfill all the requirements demanded for commercial applications, indicating that the molecular design of functionalized NIPUs remains an ongoing challenge. Future research should therefore focus on incorporating polar or positively charged moieties—such as quaternary ammonium salts or permanently charged nitrogen groups—or hybrid combinations thereof, to simultaneously optimize cell interactions, structural stability, and antimicrobial performance.

Overall, the findings of this review reaffirm that functionalization represents the most effective and sustainable way to consolidate NIPUs as advanced materials with potential for expansion into industrial and biomedical applications where high-performance and low-toxicity polymers are required. While several functionalized NIPUs show promising mechanical, physical, and biological profiles, their transition toward real applications will ultimately depend on whether future studies address aspects beyond molecular design—such as process robustness, regulatory compliance, and compatibility with scalable manufacturing routes. These considerations, although not extensively explored in the current literature, will be essential to bridge the gap between laboratory scale innovation and the industrial or clinical implementation of functionalized NIPUs.

## Figures and Tables

**Table 1:** Summary of polymer synthesis and modification techniques.

Authors	NIPUs		Synthesis Technique	Modification		Modification Technique	Form of the Material
	Carbonates	Amines		Chemistry	Physics		
[5]	Cyclic carbonated soybean oil (CSBO) (from ESBO).	Ethylenediamine (EDA).	The synthesis was carried out through the reaction of CO_2_ with epoxidized soybean oil (ESBO) in a high-pressure reactor. In this process, 250 g of ESBO and tetrabutylammonium bromide (TBAB, 8.8 wt% relative to ESBO) were added to the reactor, and the mixture was heated to 120 °C under continuous stirring. CO_2_ was then introduced into the reactor at a constant pressure of 20 bar, maintained for 24 h. After completion of the reaction, a light brown viscous oil was collected at 60–70 °C.	TiO_2_ nanoparticles (TNPs).	N/A	CSBO was weighed, and stannous octoate was added as a catalyst at room temperature. Subsequently, different concentrations of nanoparticles (NT-X, where X = 0%, 0.25%, 0.5%, and 1%) were incorporated into the formulation. The mixture was subjected to ultrasonication for 30 min, followed by the addition of a solvent mixture to reduce the viscosity of the NT-X dispersions. Finally, the curing agent (EDA) was added at 13 wt% relative to CSBO and thoroughly mixed. The films were cast into silicone molds and cured at 80 °C for 24 h, followed by an additional 4 h at 110 °C.	Films
[9]	Cyclic carbonated soybean oil (CSBO).	Tetraethylenepentamine (TEPA).	In a three-neck round-bottom flask, equimolar amounts of CSBO (6.00 g) and TEPA (1.64 g) (1:1 molar ratio of primary amine to cyclic carbonate) were introduced, followed by the addition of DMF (8 mL) to adjust the solid content to 50 wt%. The reaction temperature was raised to 70 °C under continuous stirring and maintained for 24 h. The resulting mixture was then transferred to a Teflon mold and placed in an oven at 90 °C for 12 h to allow solvent evaporation. The obtained membranes were subsequently immersed in 70% ethanol solution for 6 h to remove residual chemicals.	N/A	Azetidinium groups.	The temperature was reduced to room conditions and an appropriate amount of ECH (1.24 g or 2.48 g) was added to obtain a molar ratio of between ECH and the secondary amine groups of TEPA was 1:1 for NIPU-Az-1 and 2:1 for NIPU-Az-2. (ECH converts the secondary amine into an azetidinium cation, a positively charged group bound to the polymer). After heating the mixture at 90 °C for 12 h, polyurethane membranes functionalized with azetidinium groups were obtained.	Membranes
[8]	Thegallic acid-based cyclic carbonate.	Diamines: ethylenediamine (EDA), hexamethylene diamine (HMDA), isophorone diamine (IPDA) and Jeffamine (D230).	The reaction was carried out involving a gallic acid–based cyclic carbonate and the functionalizing compound, which were dissolved in DMF. Subsequently, different amines were added, and the mixture was stirred for 5 min to ensure homogeneity. The resulting solution was cast onto tin or Teflon plates and subjected to vacuum drying in an oven at 100 °C for 8 h, yielding dry coatings.	Polyhedral oligomeric silsesquioxanes (POSS).	N/A	The synthesis of the functionalization was performed through the incorporation of variable proportions of epoxy-functionalized POSS into the NIPU formulation. Both compounds were dissolved in the same DMF phase and underwent the same thermal curing process, thus obtaining NIPU/POSS coatings.	Coatings
[3]	Poly(propylene glycol) bis(cyclic carbonate) (PPG bisCC) and poly(tetra hydrofuran) bis(cyclic carbonate) (PTHF bisCC).	4-((4-aminocyclo hexyl)methyl)-cyclohexanamine (MBCHA) N′,N′-bis(2-aminoethyl)ethane-1,2-diamine (TAEA).	PPG bisCC (468 g/mol, 1 equivalent) or PTHF bisCC (850 g/mol, 1 equivalent) was mixed with 4-((4-aminocyclohexyl)methyl)cyclohexanamine (MBCHA, 0.2 equivalents) and N′,N′-bis(2-aminoethyl)ethane-1,2-diamine (TAEA, 0.53 equivalents), and the mixture was magnetically stirred at 40 °C for 15 min without the use of any catalyst. The solution was then poured into a flat Teflon mold (0.5 × 5 × 25 mm) and subjected to thermal curing in an oven at 70 °C for 24 h.	N/A	Poly(ethylene terephthalate) (PET) mesh.	A PET mesh (Dacron 3002, Surgical Mesh™) was placed in the mold to improve the mechanical strength of the valves. Subsequently, the liquid-state polymer was injected into the mold and allowed to polymerize and solidify at high temperature (24 h at 70 °C).	Patches
[4]	Propylene carbonate (PC)	Ethylenediamine (EDA)	PC (30.87 g, 0.30 mol) was weighed into a beaker, and EDA (9.02 g, 0.15 mol) was added dropwise to the PC solution. No solvent or catalyst was used. The mixture was magnetically stirred for 3 h at room temperature, and the resulting white solid was dried at 60 °C in a vacuum oven for 24 h. The obtained product was then ground with a kitchen grinder to achieve a particle size of 120 μm.	N/A	Carboxymethyl cellulose	Glycerol was used as a plasticizer in different proportions (90:10, 80:20, and 70:30) by using a solution casting method. A solution was prepared by adding 1 g of glycerol to 100 mL of water, followed by the gradual addition of CMC under stirring at 90 °C for 30 min. Subsequently, dry NIHU powder was incorporated at 10, 20, and 30 wt%. Stirring was maintained for an additional 30 min at 90 °C, after which the dispersion was cooled to room temperature while maintaining agitation. The mixture was then subjected to ultrasonication for 4 min at 70% amplitude. Finally, 15 mL of the dispersion was transferred to a Petri dish and dried at room temperature for 48 h.	Films
[11]	Propylene carbonate (PC)	Isophorone diamine (IPDA)	Propylene carbonate (30.00 g, 0.29 mol) was added to a 250 mL flask equipped with mechanical stirring and a reflux condenser. The mixture was heated to 120 °C, and isophorone diamine (27.52 g, 0.16 mol) was added dropwise under stirring for 8–10 h. The reaction product was cooled, dissolved in 100 mL of dichloromethane, and stirred for 1.5 h, after which 300 mL of n-hexane was added to extract the product. The resulting white precipitate was washed with n-hexane to completely remove the ammonium byproduct and any unreacted triethylamine. Finally, the mixture was dried under vacuum at 60 °C to eliminate residual n-hexane.	N/A	Polyethylene glycol diacrylate (PEGDA)Methacryloyl chloride (MAC)	The NIPU prepolymer (60.00 g, 0.16 mol) and triethylamine (36.65 g, 0.36 mol) were dissolved in 200 mL of anhydrous dichloromethane and cooled to 0 °C in an ice bath. A solution of acryloyl chloride (38.71 g, 0.37 mol) in 100 mL of anhydrous dichloromethane was then added dropwise under stirring. The reaction mixture was allowed to warm to room temperature, and after 12 h, triethylamine hydrochloride salts were filtered off. Subsequently, a saturated sodium bicarbonate solution was added. PTZ (0.05 wt%), the organic solvent was removed by rotary evaporation to obtain NIPUMA. A NIPUA solution containing the NIPUMA monomer, TEGDA, PEGDA, and 1 wt% of the photoinitiator CQ was then prepared, stirred at 50 °C and 400 rpm for 15 min, and printed.	Resin
[16]	Furan-based bis(cyclic carbonate) (FBC)	1,2-ethanediamine (EDA) and 1,6-hexanediamine (HDA)	A 100 mL three-neck flask was equipped with a mechanical stirring device and linked with a Schlenk line. The flask was then charged with 10 mmol FBC and 10 mmol diamines. The mixture was allowed to react at 60 °C for 20 min, then 80 °C for 1 h, and 100 °C for another 1 h under an inert gas atmosphere.	N/A	(Poly(propylene carbonate)	Before blending, PPC was dried in a vacuum drying oven at 30 °C for 24 h. Then, it was melting blended with furan-based NIPUs by an internal mixer under 110 °C for 10 min. The weight ratio of PPC/NIPU was set as 97.5/2.5, 95/5, 92.5/7.5, 90/10, and 85/15. To prepare the sample sheets for measurements, PPC and its blends were compression-molded at 110 °C under 10 MPa.	Sheet
[18]	Ethylene carbonate (EC)	1,6-hexanediamine (1,6-HDA)	EC and 1,6-HAD were used as raw materials for the synthesis of BHHDC by heating the reaction at 80 °C for 2 h and then at 96 °C for another 2 h. Subsequently, 180 g of deionized water was added, and the reaction was maintained at 80 °C for 30 min before cooling the mixture to room temperature. This BHHDC was then used with PEG1000 for the synthesis of NIPU, employing SnCl_2_·2H_2_O as a catalyst. The reaction temperature was 220 °C under a pressure of 600 Pa.	N/A	Low-molecular-weight polyglycolic acid diol (LPGD) and curcumin	LPGD171 and NIPU were used as raw materials for NI-LPGD synthesis, with HFIP serving as the solvent. The reaction was conducted at a temperature of 60 °C for 30 min. Among the NI-LPGD, the mass fraction of LPGD171 was from 5 wt% to 20 wt%, and they were named NI-LPGD5, NI-LPGD10, NI-LPGD15 and NI-LPGD20.	Membranes
[17]	Polycarbonate diols (PCDLs)	1,6-hexanodicarbamato (1,6-HDC)	They were synthesized by the transurethane reaction of PCDL and 1,6-HDC. PCDL (43.0 mmol), 1,6-HDC (43.0 mmol), and TBT (0.2–0.3 wt%) were added to a 100 mL round-bottom flask equipped with a condenser and a mechanical stirrer. The mixture was heated to 170 °C and mechanically stirred until a homogeneous solution was obtained. Subsequently, the reaction mixture was transferred to a vacuum oven and heated at 170 °C under dynamic vacuum. The product was dissolved in DMF (80 mL) at 70 °C, precipitated in methanol (2 L), and dried in a vacuum oven at 40 °C overnight. Finally, an electrospinning process was carried out.	N/A	Rat tail collagen, type I (rCol I).	Collagen was extracted from rat tail tendons using an acid-based isolation method and lyophilized for long-term storage. Prior to biofunctionalization, the lyophilized collagen was solubilized in acetic acid (0.1 M) at a concentration of 0.1 mg/mL. The sheets were incubated in the collagen solution at 37 °C for 2 h, the excess solution was aspirated, and they were left to dry at 4 °C overnight.	Scaffolds
[19]	Trimethylolpropane monoallyl ether cyclic carbonate (TMPMEC)	Cadaverine (1,5-diaminopentane, >99%)	TMPMEC and cadaverine were mixed at a molar ratio of 2:1 in order to accommodate ring-opening conjugation with cadaverine’s two available primary amines. This mixture of TMPMEC and cadaverine was initially vortexed vigorously for 1 min and then heated (68°C for 15 min), during which it was also periodically vortexed.	N/A	Thiol-ene polymerization of alkene groups	Various molar ratios of thiol compounds were introduced to match the number of alkenyl groups in the diallyl-diurethane prepolymer. The mixtures were then heated at 78 °C for 5 min. Following this step, Darocur 1173® (2% *v*/*v*) was added, and the mixtures were stirred intermittently for an additional 5 min at 78 °C.	Slabs
[20]	Carbonated sunflower oil (CSFO)	Polyamine polyol (PAPO)	The CSFO and PAPO monomers were reacted at a molar ratio of 1:1.5, considering their functionality. After homogenization by mechanical stirring at 90 °C for 1–3 min, the viscous solution was poured into silicone molds and maintained at 90 °C for 24 h to promote crosslinking.	N/A	Acrylate Acid grafted LbL deposition of chitosan and alginate TTO	NIPU films were placed in Petri dishes containing a solution of BP dissolved in AA (0.5 mL, 0.2 M) and distilled water (0.5 mL). This system was exposed to UV light (365–415 nm) for 30 min. The samples were then thoroughly washed with NaOH solution followed by distilled water to remove any residues. The NIPU films with grafted AA (AANIPU) were vacuum-dried at room temperature until constant weight was achieved, after which a second treatment was carried out with AL-CS or TTO. A multilayer coating of cationic chitosan (CS) and anionic alginate (AL) was deposited onto the AANIPU films using the layer-by-layer (LbL) assembly method. Other treatment, AANIPU films were immersed in a TTO solution (50 wt%) for 24 h at 4 °C, using acetone as solvent. The samples were rinsed with acetone to remove excess TTO, washed with distilled water, and dried at room temperature until complete solvent evaporation.	Films
[21]	Poly(ethylene glycol) Bis-cyclic Carbonate (PEGC)	Triethylenetetramine	To a three-necked bottom flask equipped with a refluxing condenser, the requisite amounts of PEGC and TEDA (at a 1:1molar ratio) were added and stirred while being heated to 50 °C. Subsequently, a mixture of TETA and methanol was added to the flask in three equal parts over three half-hour intervals using a syringe, and the reaction was allowed to proceed for 4 h at 50 °C. After the reaction was completed, the condenser was removed from the reaction system, and the methanol was allowed to evaporate completely.	N/A	Gelatin Epoxidized Graphene Oxide (EGO) Ag NPs	EGO powder was added to a 50 mL beaker containing 15 mL of deionized water, and the mixture was stirred at room temperature for 3 h using a magnetic stirrer. The nanoparticles were dispersed in water using ultrasound for 10 min. Solutions were prepared with the specified amounts of GE, PHU, and BDDE in separate beakers, with 10 mL, 10 mL, and 5 mL of deionized water, respectively. This was then added to the EGO solution, stirred for 15 min with a magnetic stirrer, and subjected to ultrasound for 10 min. It was slowly poured into a silicone mold and kept at 40°C for 24 h, finally undergoing additional heat treatment in a vacuum oven at 80°C for 36 h. On the other hand, the PHU-G5 and PHU-G-EGO2 membranes were immersed in a beaker with silver nitrate solution (1% by weight) and left in the dark for 36 h. They were then removed and washed with plenty of distilled water. Finally, the membranes were dried at room temperature under vacuum.	Films
[22]	Cyclic carbonated soybean oil (CSBO)	N, N′-Dimethylethylenediamine (DMEDA)	NIPU-TA was synthesized by reacting CSBO and DMEDA, following the procedure described by Yeganeh et al. [22] (A round-bottomed flask was charged with CSBO (100.00 g, 0.29 mol), LiCl (1.20 g, 0.028 mol) and THF (80 mL). DMEDA (33.23 g, 0.377 mol) was then added to the flask and the resulting solution was stirred at room temperature. At the end of the reaction, the flask content was diluted with ethyl acetate and extracted twice with distilled water slightly acidified by adding hydrochloric acid. The organic layer was then separated and freed from dissolved water via treatment with dry sodium sulphate).	N/A	(3-chloropropyl) trimethoxysilane (CPTMS), (3-Glycidyloxypropyl) trimethoxysilane (GPTMS), Tetraethyl orthosilicate (TEOS) and gelatin	A three-necked round-bottomed flask equipped with a condenser, a magnetic stirrer, a thermometer, and a nitrogen inlet was charged with 5.0 g NIPU-TA, 2.0 g CPTMS, and moisture-free DMF solvent. The reaction mixture was stirred at 85 °C for 48 h. Finally, the solvent was evaporated, and the final product was subjected to high vacuum in an oven at 80 °C. In a beaker equipped with a magnetic stirrer, 5.0 g of QMSiNIPU, 0.5 g of TEOS, and 1.5 g of GPTMS were completely dissolved in 10 mL of DMF at room temperature. Then, three drops of acetic acid and one drop of Sn(oct)2 were added to the solutio and was slowly poured into a silicone mold. This was placed in an oven at 80 °C for 12 h and then at 120 °C for 2 h. The GE solution with a concentration of 4 wt%. Then, the NIPU/Si was immersed in the as-prepared solution at the same temperature, and the reaction was continued for 12 h. Finally, the membrane was thoroughly washed multiple times with an ample amount of double-distilled water. The resulting film was then placed in a vacuum oven at 60 °C.	Membranes
[23]	Poly(propylene glycol)-bis(cyclic carbonate) (PPG bisCC)	4,4’-methylenebis(cyclohexylamine) (MBCHA)	Was synthesized by the solvent- and catalyst-free polyaddition of poly(propyleneglycol)-bis(cyclic carbonate) (PPG bisCC, synthesized from a 380 g/mol PPG-diglydicylether precursor) with 4,4’-methylenebis(cyclohexylamine) (MBCHA) at 70 °C for 96 h, as already reported in the literature.	N/A	α-alkylidene cyclic carbonate (αCC) Thiol–ene reaction	Five grams of PPG-PHU were dissolved in dry DMF (20 mL) in a 100 mL round-bottom flask equipped with a magnetic stirrer. After complete dissolution, αCC and 1,8-diazabicyclo[5.4.0]undec-7-ene (DBU) were added under an inert atmosphere and stirred for 24 h. The product was purified by three successive precipitations in diethyl ether, centrifuged (10,000 rpm, 10 min, 15 °C), redissolved in CHCl_3_, and finally dried under vacuum. For photochemical crosslinking, the αCC-modified NIPU containing pendant C=C bonds was mixed with polythiols (SH_2_, SH_3_, or SH_4_) and Irgacure 819, followed by UV irradiation to trigger the thiol–ene reaction.	Resin
[24]	Adipate bicarbonate or alkyl C10 diglycerol carbonate	1,8-diaminooctane	For the synthesis of the polyurethane capsules, 83 mg (0.57 mmol) of 1,8-diaminooctane, 1.0 g of water, 1.0 mg of dye or doxorubicin and 6.0 mg sodium chloride were added to 7 g of cyclohexane containing 200 mg of Hypermer™ B246. For pre-emulsification, the reaction mixture was stirred at room temperature for 1 h at 1200 rpm. After that, the mini-emulsion was obtained by ultrasonication of the mixture for 3 min. An equimolar amount of bis carbonate moiety with respect to the amino monomer was dissolved in 4 g of the cyclohexane-dichloromethane mixture. To this, a catalytic amount of TEA was added. The reaction mixture was added in a dropwise manner to the above mentioned mini-emulsion dispersion, and the resulting mixture was left for stirring at room temperature 24 h.	N/A	Post-grafting of the nanocapsules with phosphonium ion	Briefly, 2.0 g of NIPU nanocapsule dispersion (solid content of 5.0 wt %), (5-carboxypentyl)triphenylphosphonium cation (0.1 g), and 4- dimethylaminopyridine (0.05 g) were dissolved in 10 mL of dry DCM. N-ethyl-N׳-(3-dimethylaminopropyl)carbodiimide hydrochloride (EDC.HCl) (0.08 g) was dissolved in CH2Cl2 (1 mL) and added dropwise to the reaction mixture at 0 °C with stirring. The reaction mixture was allowed to for a8 h at room temperature, and then the nanocapsule solution was transferred into SDS water solution. The resulting dispersion was stirred at 1000 rpm for 2 h at room temperature. Subsequently, the reaction mixture wasultrasonicated for 10 min. This was left to stir overnight at 1000 rpm at room.	Nanocapsules
[25]	4,4′-(((furan-2,5-diylbis(methylene)) bis(oxy)) bis (methylene)) bis(1,3-dioxolan-2-one) (FuBCC) and bis((2-oxo-1,3-dioxolan-4-yl)methyl) succinate (SuBCC)	1,5-pentanediamine (cadaverine)	FuBCC (10.3 g, 31.5 mmol) or SuBCC (10.0 g, 31.5 mmol) was added into the reactor. The content was dissolved in anisole (30 mL), and diamine (3.218 g, 31.5 mmol) was added. The reactor was sealed, and the reaction mixture was stirred for 28 h at 70 °C. Finally, the reaction mixture was cooled down to room temperature and the excess anisole was decanted. The polymer was then dissolved in methanol (10 mL) and the polymer was precipitated from diethyl ether (20 mL). The process was repeated two more times. The polymer was dried under air and then under vacuum at 80 °C, giving poly(FuCa) and poly(SuCa) in yields above 90%.	N/A	Post polymerization functionalization of NIPUs by phosphorylation	To a solution of poly (FuCa) in GVL (25 mL), trichloroacetonitrile (TCAN) was added followed by dropwise addition of a solution of tetrabutylammonium dihydrogen phosphate (TBAP) in anhydrous acetonitrile (10 mL). After the addition, the reaction mixture was allowed to stir for 18 h at 50 °C. Upon completion of the reaction, the solvent was removed The solid was then dissolved in a small amount of methanol (10 mL) and re-precipitated using diethyl ether (50 mL). This step was repeated three times before drying under vacuum to obtain poly(FuCa)-P15. To a solution of poly(FuCa) in GVL (25 mL), TCAN was added followed by dropwise addition of a solution of TBAP in anhydrous acetonitrile (50 mL). After the addition, the reaction mixture was allowed to stir for 18 h at 50 °C. Upon completion of the reaction, the solvent was removed to obtain. The solid was then dissolved in a small amount of methanol (10 mL) and re-precipitated using diethyl ether (50 mL). This step was repeated three times before drying under vacuum to obtain poly(FuCa)-P50.	Multifunctional additives

**Table 2:** Improved physical and mechanical properties of functionalized NIPUs.

Authors	Tensile s.	Young’s Modulus	Storage Modulus	Elongation at Break	Water Contact Angle	Absorption of Water	Water Vapor Transmission Rate WVTR	Glass Transition Temperature (Tg)	Thermal Stability
[5]	NT-1: (↑) 2,28 ± 0,11 MPa	N/A	NT-1: (↑) 2207 MPa	NT-0.25: (↓) 162.9 ± 13.7%	NT-1: (↑) 105.4 ± 1.3°	N/A	N/A	NT-1: (↑) 2769 °C	NT-1: (↑) 480.42 °C
[9]	NIPU-Az-2: (↑) 6.21 ± 0.18 MPa	NIPU-Az-2: (↑) 41.1 ± 4.7 MPa	NIPU-Az-2: (↑) (2638.6 MPa)	NIPU-Az-2: (↓) 223 ± 7%	NIPU-Az-2: (↓) 60.34 ± 0.49°	NIPU-Az-1: (↑) 37.55%	NIPU-Az-1: (↑) 896 gr /m^2^/day	NIPU-Az-2: (↑) 60 °C	N/A
[8]	N/A	N/A	N/A	N/A	N/A	NIPU/POSS (4): (↓) 23.38%	N/A	NIPU/POSS(3): (↑) 57.58 °C	NIPU/POSS(1): (↑) 388.22 °C
[3]	PPG-NIPU + PET (Dry): (↑) 1.79 ± 0.10 MPa		PPG-NIPU + PET (Dry): (↑) 73.00 ± 19.51 MPa	PPG-NIPU + PET (Wet): (↑) 83.9 ± 31.9%	N/A	PPG-NIPU + PET: (↑) 36.76%	N/A	N/A	N/A
[4]	CMC/NIHU2: (↓) 22.03 MPa	CMC/NIHU1: (↓) 1017.50 MPa	N/A	CMC/NIHU3: (↑) 17.2%	CMC/NIHU3: (↑) 70.0°	N/A	N/A	CMC/NIHU1: (↑) 162.7 °C	CMC/NIHU1: (↑) 207.9 °C
[11]	PEGDA 16%: (↑) 63.93 MPa	PEGDA 12% (↓) ± 2100 MPa	PEGDA 16%: (↑) < 5 GPa	(↑) 59.2%	N/A	N/A	N/A	N/A	PEGDA 20% (↑) 253.92 °C
[16]	>(↑) 30 MPa	N/A	N/A	PPC/NIPU 1c and PPC/NIPU 2c: Increase 10%	N/A	N/A	N/A	PPC/NIPU 2c: (↑) 34.5 °C	(↓) Lees than 200 °C
[18]	Cur/NI-LPGD5: (↑) 5.0 ± 0.4 MPa	N/A	N/A	(↓) 36.1 ± 5.5 %	NI-LPGD20: (↓) 44°	N/A	NI-LPGD5: 3405.0 ± 24.1 g/(m^2^·d)	NI-LPGD: (=) −40 °C	NI-LPG: (↑) 272 °C
[17]	N/A	N/A	N/A	N/A	Biofunctionalized NIPU: (↓) 98° ± 2	N/A	N/A	NIPU-C: (↑) −41 °C	N/A
[19]	N/A	NIPU-4SH 100%: 2500 MPa	NIPU-4SH 100%: 1.2E Pa	N/A	N/A	N/A	N/A	NIPU:50%4SH–50%3SH: −0.73 °C.	250 °C
[20]	AL-CSNIPU: (↓) 591.99 ± 130.3 kPa	AL-CSNIPU: (↑) 7400 ± 6.8 kPa	N/A	AANIPU: (↓) 127.0% ± 0.26	AL-CSNIPU: (↓) 79.77°	N/A	N/A	AL-CSNIPU: (↓) −17.68 °C	T75% (°C) AL-CSNIPU: (↑) 380.28
[21]	PHU-G-EGO2: Dry (↑) 17.22 ± 0.20 MPa	PHU-G-EGO2: Dry (↑) 233.37±9.04 MPa	PHU-G-EGO2: (↓) 2.74 MPa	PHU-G-EGO2: Dry (↓) 116.66 ± 12.67 %	N/A	PHU-G-EGO1: (↓) 160.87±2.25%	PHU-G-EGO1: (↓) 1.45 ± 0.02 (g 10^–1^ cm^−2^ day^−1^)	PHU-G-EGO2: (↓) −2.96 °C	N/A
[22]	NIPU/Si-GE (dry): (↑) 7.91 ± 0.74 MPa	NIPU/Si-GE (Dry): (↑) 88.90 ± 4.67 MPa	NIPU/Si-GE: (↑) 2.06 ± GPa	NIPU/Si-GE (Dry): (↓) 12.90 ± 3.27%	NIPU/Si-GE: (↓) 78.70 ± 1.92°	NIPU/Si-GE: (↑) 69.2 ± 2.1%	NIPU/Si-GE: (↓) 2.48 ± 0.04 (g 10^−1^ cm^−2^ d^−1^)	NIPU/Si-GE: (↑) 17.7 °C	N/A
[23]	SH4: Dry: 1.1 ± 0.1 MPa	SH4: Dry: 16.0 ± 1.0 MPa	SH4: 58,000 Pa	SH2: Dry: 108.6 ± 1.3%	N/A	SH2: 4.2 ± 0.5%	N/A	SH4: 42.4 °C	N/A
[24]	N/A	N/A	N/A	N/A	N/A	N/A	N/A	N/A	N/A
[25]	N/A	N/A	N/A	N/A	N/A	N/A	N/A	poly(FuCa)-P15: (↑) 21 °C	poly(FuCa)-P15: (↓) 183 °C

(↑) Indicates an increase and (↓) indicates a decrease, as reported by the authors relative to their respective controls. Cells without arrow correspond to studies that did not include a control group or did not report comparative data.

**Table 3:** Improved biologicals properties of functionalized NIPUs.

Authors	Antimicrobial Activity	Cell Viability	Biocompatibility	Hemocompatibility
[5]	NT-1: 3 mm (*E. coli* MTCC 443)	N/A	N/A	N/A
[9]	NIPU-Az-2: 100 ± 0% (*E. coli*) ATCC 25922 98.27 ± 0.41% (*S. aureus*) ATCC 6538	85% in L929 mouse fibroblast cells	N/A	N/A
[8]	N/A	N/A	N/A	N/A
[3]	N/A	PPG-NIPU Human primary fibroblasts: 78.7 ± 4.5% (HUVECs): 87.4 ± 5.0%	N/A	Only PPG-NIPU and PTHF-NIPU: −0.2 ± 0.15%
[4]	N/A	N/A	N/A	N/A
[11]	N/A	Murine fibroblast L929 > 90%	Bone Cells (MC3T3-E1) and Muscle Cells (C2C12) absence of an inhibitory effect	>0.5%
[16]	N/A	N/A	N/A	N/A
[18]	*E. coli* (ATCC 8739) was not significant *S. aureus* (ATCC 29213) > 90%	Murine fibroblast L929 > 98%	N/A	Hemostatic capacity in vivo: NI-LPGD5: (↓) 125 ± 37 mg
[17]	N/A	L929 > 70%	MeT-5A cells adverse effects on cell proliferation	N/A
[19]	N/A	Murine myoblasts: cell death (−3%)	N/A	N/A
[20]	*S. aureus* (ATCC 25923) AA NIPU and AA_TTO NIPU *E. coli* (ATCC 25922) AA_TTO NIPU	Detroit 551-CCL 110™ fibroblasts. AANIPU: 73.94 ± 6.59 % TTONIPU: 46.32 % ± 4.05	N/A	N/A
[21]	*Staphylococcus aureus* (ATCC 6538): 92.8 ± 0.9% *E coli*: 89.3 ± 0.8 %	L929 mouse fibroblast cell: >80%	N/A	N/A
[22]	*S. aureus,* (ATCC 6538): 82.2% *E. coli*, (ATCC 25922): 50.2%	L929 mouse fibroblast cells: >80%	N/A	IPU/Si-GE: <0.5%
[23]	N/A	Human fibroblasts: >80%	N/A	PPG-NIPU con SH2: 0.3%
[24]	N/A	LN229 cells: Nontoxic up to 100 micromolar concentrations	N/A	N/A
[25]	*Bacillus* (ATCC 35889): poly(FuCa)-P50: 21.7 mm *E. coli* strain K12: poly(FuCa)-P50: 24.8 mm	Human keratinocyte HaCaT cells: >80% in most concentrations	N/A	N/A

(↓) indicates a decrease.

**Table 4:** Applications and main results of the studies of functionalized NIPUs.

Authors	Application Type	Study Design	Results Mainly	Methodological Limitations
[5]	Eco-friendly adhesives Antimicrobial coatings Protective surfaces	In vitro	In summary, the TNP-incorporated NIPU exhibited improved thermal stability, mechanical properties, hydrophobicity, and antimicrobial effectiveness compared to bare NIPU, highlighting their potential for high-performance, environmentally friendly coatingapplications.	Incomplete evaluation of properties, due to the absence of Young’s modulus, water absorption, among other relevant tests. Since their intended use is as antimicrobial coatings, only two samples of each material type were tested to obtain an average value for each group (reduced number of replicas). And finally the antimicrobial assays showed limited results.
[9]	Wound dressing membranes	In vitro	The combination of hydrogen bonding, azetidinium ionic interactions, and C-N covalent crosslinking resulted in excellent dry strength, extensibility, and dimensional stability upon hydration. Therefore, these compounds are expected to protect injured tissue, maintain an optimal humid environment, and balance water retention and vapor transmission, for later use as an advanced wound dressing.	Absence of hemocompatibility assays, despite the material being intended for use as wound dressing membranes. Lack of chemical resistance testing, considering that the document reports that cyclic-carbonate-derived polyurethanes may exhibit insufficient mechanical properties and chemical resistance to aqueous acid and base solutions. Finally, the absence of in vivo assays prevents confirmation of their practical applicability.
[8]	Coatings	In vitro	Compared to NIPUs without POSS addition and those with POSS addition, NIPUs/POSS showed greater pencil hardness, water resistance and thermal stability, but lower adhesion; however, water absorption decreased and impact resistance and flexibility were affected only in NIPUs 3.	Lack of all biological evaluations, despite being a material derived from a renewable resource no assays on biocompatibility or biodegradability were performed. Assessment of mechanical properties remains incomplete, even though the material is intended for use as coatings.
[3]	Prosthetic heart valve	In vitro	The study lays the foundation for the future development of prosthetic heart valves made from NIPUs that are hemocompatible and hemodynamically competent, with good mechanical, elastic and stable properties, low hemolytic behavior, low platelet adhesion, resistance to calcification and excellent cytocompatibility with fibroblasts and endothelial cells.	No studies were conducted on chemical degradation resistance or biodegradation. In addition, the incorporation of the PET mesh may affect the interaction with blood components. There are difficulties in achieving optimal valve opening, durability tests are required. Finally, the absence of in vivo assays prevents confirmation of their practical applicability.
[4]	Packaging	In vitro	In particular, the incorporation of 20% (*w*/*w*) NIHU into CMC significantly improved the mechanical properties of resulting hybrids. The versatility and simplicity of the synthesis of NIHU and its hybridization with CMC, and the overall improvements in mechanical, thermal, and structural properties, these hybrids can make them promising candidates for many packaging applications.	Since this material is intended for packaging applications, biocompatibility and biodegradability tests were not performed. Furthermore, some properties were only compared to those of pure CMC, and not to those of pure NIHU.
[11]	3D printing of custom biocompatible orthopedic surgical guides	In vitro In vivo	By adding 12% of PEGDA weight, it was possible to achieve maximum tensile and flexural strength, better elongation, thermal stability, resistance to acid/alkaline corrosion and excellent biocompatibility, taking into account cell viability, cell proliferation, among other things, with commercial materials it demonstrates lower toxicity and greater safety, which highlights its potential as a renewable medical-grade photocurable resin for 3D bioprinting and clinical applications.	Incomplete curing occurred during the process, and insufficient results were obtained regarding the corrosion resistance of NIPUA, since no quantitative measurements of mass change or residual mechanical properties were reported.
[16]	High-performance biodegradable polymeric materials	In vitro	Thanks to the abundant N―H and O―H groups in NIPU and the C=O groups of PPC, strong intermolecular interactions through hydrogen bonding were formed, and the glass transition temperatures, tensile strength, and elongation at break were significantly improved. In particular, by adding 5.0 wt% of NIPU, the tensile strength of the blends reached values above 30 MPa, approximately twice that of pure PPC and potentially equivalent to that of commercial polyethylene. This work demonstrates the preparation of high-performance, sustainable, and optimally biocompatible CO_2_-based biodegradable polymer materials.	Biological tests are lacking to confirm the intended purpose of the material as both biodegradable and biocompatible.
[18]	Wound dressing	In vitro In vivo	Based on our results, it can be concluded that the Cur/NI-LPGD5 nanofibrous membrane possesses excellent mechanical properties, water vapor transmittance, antibacterial properties, biocompatibility, and hemostasis ability; furthermore, it can effectively prevent chronic wound development and can serve as a wound dressing without requiring frequent replacement.	The presence of fluorine in the materials makes it necessary, in future studies, to identify a solvent with high polarity, volatility, and no hydroxyl groups for the synthesis process of the NI-LPGD membrane. A broader antibacterial evaluation against multidrug-resistant pathogens is also required, and finally, it is important to increase the number of experimental subjects in future investigations.
[17]	Substitute in cardiac tissue engineering	In vitro	Despite high water contact angles, the bare electrospun NIPU mats did not underperform in comparison to collagenfunctionalized mats because both fibroblasts and epithelial cells displayed good adhesion and proliferation. These in vitro investigations of the electrospun NIPU mats showed that they bear great potential in biomimetic cardiac scaffolds.	Further studies are needed to evaluate the mechanical properties of this material. Additionally, the use of a fluorinated solvent for solubilizing the NIPU represents a limitation, as it poses safety risks. A comparison with a commercially available TPU is necessary to confirm its functionality. Finally, the lack of in vivo testing prevents confirmation of its practical applicability.
[19]	Biomedical engineering (3D printable devices)	In vitro	The physical properties of NIPUs made from different combinations of linear and branched thiols were characterized, and they were shown to be tunable, cytocompatible, and printable by light-based 3D printing, making them novel, biocompatible, and mechanically flexible. The tunability and characteristics of the printable NIPU materials can be improved by using other types of biogenic polyamines, such as putrescine (C4 diamine), spermidine (triamine), and spermine (tetraamine), instead of cadaverine (C5 diamine).	The material exhibits low cell adhesion, which may represent both advantages and disadvantages, making it necessary to achieve a balance in this specific property. In addition, in vivo studies are required to demonstrate its potential use as 3D printable devices.
[20]	Antibacterial coating	In vitro	Its hydrophobic surface does not provide the antibacterial behavior expected for biomedical applications. A radical UV-initiated modification was performed to graft AA onto the surface and incorporate two antibacterial agents. Only TTO showed antibacterial activity against *E. coli* and *S. aureus*, but all surface modifications reduced the mechanical properties of the films. Further research is needed to understand the surface interactions between the different compounds and the nitrile protective film (NIPU).	The results indicate that the materials may potentially be considered cytotoxic. Moreover, contradictions were observed in some properties, such as the Tg versus surface stiffness and flexibility, which could not be fully understood due to experimental limitations. Finally, quantification of alginate and chitosan is necessary, since no antimicrobial activity was observed under the evaluated conditions.
[21]	Antibacterial wound dressings	In vitro	The strength of this sample was improved to 17.22 and 0.79 MPa (dry and wet conditions) by adding 2% by weight of EGO. The presence of Ag nanoparticles in the backbone of the dressings provided good antibacterial activity against various bacterial strains without severe cytotoxicity toward fibroblasts. This characteristic was enhanced to 90% bacterial elimination in samples containing Ag nanoparticles and EGO.	Lack of hemocompatibility tests, given that the material is developed as a wound dressing, and the absence of in vivo studies to evaluate its effectiveness.
[22]	Antibacterial wound dressing membrane	In vitro	The results indicated that the dressings exhibited excellent blood compatibility, attributed to the balanced concentration of QAS moieties within their structure and the appropriate level of water absorption, which effectively aids in the absorption of proteins involved in the blood clotting process. Overall, the properties of the developed dressings make them highly suitable for protecting low to moderate-exuding wounds, as well as infected wounds.	The results showed increased platelet adhesion, which highlights the need to achieve stability in this property. In addition, only limited activity against Gram-negative bacteria was observed, and finally, in vivo tests are still required to confirm the effectiveness of the material.
[23]	Applications in the cardiovascular field.	In vitro	The impact of the polythiols used on the thermomechanical properties of the final materials showed adequate values for various biomedical applications. In addition, in vitro biocompatibility/hemocompatibility tests, performed in contact of the materials with human fibroblasts, red blood cells, human platelets or platelet-poor plasma, showed biocompatible and hemocompatible profiles of the NIPUs, demonstrating the suitability of these materials for use in a biomedical context, including implants in contact with blood.	Use of solvents classified as “2” by the FDA, which raises safety concerns. Oxidation of the material was observed, leading to a “brown” coloration, which would prevent applications where strictly colorless materials are required. Finally, the absence of in vivo testing limits its potential application in the cardiovascular field.
[24]	Nanocapsules for Enzyme-Triggered Drug Release	In vitro	The drug carriers are synthesized using the inverse mini-emulsion technique by exploiting an in situ NH2–carbonate green reaction at the droplet interface. In addition, these nanocarriers can also be post grafted with organelle-specific targeting ligands for on-demand, target specific, drug delivery and bio-imaging. These nanocarriersoffer a promising novel therapeutic platform with high potential for biological imaging and drug delivery to fight cancer and other diseases.	Lack of evaluation of mechanical properties and stability tests. In addition, in vivo studies are needed to confirm their effectiveness.
[25]	Multifunctional additives in personal care/cosmetic applications.	In vitro	The resulting NIPUs were functionalized by mild and selective phosphorylation using tetrabutylammonium dihydrogen phosphate as the phosphate source, resulting in the production of NIPU phosphate monoesters with up to 50% phosphate content and showing the following features: (1) water solubility/dispersibility, (2) characteristic aerobic biodegradability, with degradation ranging from 56% to 75% within 28 days, absence of toxicity, with minimal or no inhibition of human keratinocyte HaCaT cell growth at concentrations up to 0.5 mg mL^−1^, absence of skin.	More exhaustive in vitro and in vivo studies are needed to fully understand the toxicity profile. The E factor results could be improved by optimizing reaction conditions and purification processes through solvent reduction and recycling. In vivo evaluation is necessary to determine both efficacy and non-toxicity.

## Data Availability

The data presented in this study is available on request from the corresponding author.
